# Suppression of neuroinflammation by the dopamine receptor D2 via alphaB-crystallin

**DOI:** 10.1186/1750-1326-7-S1-L15

**Published:** 2012-02-07

**Authors:** Jiawei Zhou

**Affiliations:** 1 Institute of Neuroscience, State Key Laboratory of Neuroscience, Shanghai Institutes for Biological Sciences, Chinese Academy of Sciences, Shanghai, 200031, China

**Keywords:** dopamine receptor D2, astrocyte, alphaB-crystallin, inflammation, Parkinson's disease

## 

Chronic neuroinflammation is a common feature of aging brain and some neurodegenerative diseases, including Parkinson's disease. However, the molecular mechanisms underlying regulation of innate immunity in the central nervous system remain elusive. Here we show that dopamine receptor D2 (Drd2), known as a critical component for dopamine neurotransmission and a susceptibility gene for Parkinson's disease, modulates innate immunity through its downstream target protein astrocytic alphaB-crystallin (Cryab). We demonstrate that knockout mice lacking Drd2 showed remarkable inflammatory response in multiple CNS regions and increased vulnerability of nigral dopaminergic neurons to MPTP-induced neurotoxicity. Drd2—/— astrocytes became hyper- responsive to immune stimuli with dramatic reduction in levels of Cryab. Gain- or loss-of-function studies showed that Cryab is critical for Drd2-mediated modulation of innate immune response in astrocytes. Interestingly, aberrant expression of Cryab in reactive astrocytes was found in the ventral mesencephalon of patients with Parkinson's disease. Furthermore, treatment of wild-type mice with a selective Drd2 agonist increased resistance of the nigral dopaminergic neurons to neurotoxin via partial suppression of inflammation. Altogether, these data suggest that Drd2 / Cryab pathway in astrocytes is crucial for repression of neuroinflammation and thus could be therapeutically useful for treatment of Parkinson's disease.

